# Eye: The Hard-hit Victim of COVID-19 Pandemic

**DOI:** 10.18502/jovr.v17i2.10805

**Published:** 2022-04-29

**Authors:** Aman Gaur, Prathama Sarkar

**Affiliations:** ^1^Vardhman Mahavir Medical College & Safdarjung Hospital, New Delhi; ^2^Deen Dayal Upadhyay Hospital, New Delhi

**Keywords:** kwd

## Abstract

The COVID-19 pandemic has brought the entire world to a standstill. Wearing of mask and time-to-time sanitization have become a customary daily practice. Additionally, as the outdoor activities and movements have been curtailed, concept of work from home is being widely adopted. Hence, the screen exposure time has considerably increased. All these conditions have directly or indirectly impacted the health of eye. This article emphasizes on the repercussions of this pandemic on eye health. It also focuses on the precautions that may be taken to prevent them as well as some solutions to manage them.

##  INTRODUCTION

The COVID-19 pandemic since the end of year 2019 has not only forced the entire world into lockdown but it has also tremendously changed the way we live and work. This has given rise to various health concerns. Eye problems amidst this crisis is no more a mere possibility. But rather it has now become a reality. In this COVID era, apart from SARS-CoV-2 viral conjunctivitis, other eye-related problems are also on a rise due to sudden changes in ergonomics. As nonessential outdoor activities are being avoided, most institutes and office spaces have been abandoned and work from home has become a common practice. Conference meetings and teaching sessions have been replaced by web conferencing. With increased dependency on digital platforms, our screen exposure time has also increased. This has resulted in sudden upsurge in patients complaining of dry eyes and eyestrain.

Incessant use of sanitizers and disinfectants is giving rise to yet another problem of sanitizer aerosol-driven ocular surface disease (SADOSD).^[1]^

Also, reckless use of steroids in the management of COVID may predispose these patients to opportunistic infections,^[2,3,4,5]^ including rhino–orbital mucormycosis^[6,7,8,9,10]^ which is a serious ophthalmic concern.

Since most patients are opting for telemedicine, conducting direct eye examination and other investigations now have a limited role to play. Hence, it has become imperative to know about the changing pattern of eye diseases in the population to deliver the best possible care under these limitations.

In this article, the authors have attempted to provide the most up-to-date information regarding the new emerging pattern of eye diseases during the COVID-19 pandemic which may be helpful to ophthalmologists while providing eye consultations. Few common eye problems which may arise as a result of this pandemic are discussed below.

### Viral Conjunctivitis

Conjunctivitis may either be the first presentation in patients infected with novel COVID-19^[11,12]^ coronavirus or it may be associated with respiratory illness.^[13,14,15,16]^

In the “Report of the WHO–China Joint Mission on Coronavirus Disease 2019 (COVID‐19),” conjunctival congestion was reported in 0.8% of 55,924 laboratory-confirmed COVID-19-positive patients as per data collected in the period between February 16 and 20, 2020.^[17]^ Similarly, a meta-analysis of three studies from China including 1167 patients reported an overall rate of conjunctivitis as 1.1%. The rate of conjunctivitis was higher in severe (3%) as compared to non-severe (0.7%) COVID-19 patients with an odds ratio of 3.4.^[18]^

However, a systematic review which included 11 published articles analyzing 252 SARS-CoV-2 patients infected globally reported a variable prevalence of conjunctivitis ranging between 0 and 32%, although no meta-analysis was performed. Altogether, 17 patients had conjunctivitis of which three were tested positive for tear PCR and 14 had negative-tear PCR.^[19]^

“Pink eye” due to COVID-19 may present like any viral conjunctivitis. In a study conducted at a tertiary care hospital in Wuhan, out of 121 patients with confirmed COVID-19, 8 patients (6.6%) showed ocular symptoms which included itching (62.5%), redness (37.5%), tearing (37.5%), discharge (25%), and foreign body sensation (25%).^[13]^ There was no statistical correlation of ocular findings with duration of illness.^[13]^ A study from Hubei province in China reported ocular symptoms in 12 out of 38 COVID-19-positive patients with signs including hyperemia, chemosis, and epiphora. About one-third cases with ocular signs were observed to have severe COVID-19 manifestations. Patients with ocular manifestations had a higher white blood cell and neutrophil count along with higher levels of procalcitonin, C-reactive protein, and lactate dehydrogenase as compared to patients without ocular complaints.^[14]^ A case study from Shenzhen, China reported a patient who presented with bilateral conjunctivitis on day 13 of illness having complaints of redness, foreign body sensation, and tearing in both eyes without any blurring of vision.^[15]^ On slit-lamp examination, moderate conjunctival injection was noticed with watery discharge and inferior palpebral follicles. Bilateral preauricular lymph nodes were tender and palpable. Patient showed improvement in ocular signs and symptoms from day 15 of illness on ribavirin eye drops with complete resolution of symptoms by day 19. Conjunctival swabs collected on day 13, 14, and 17 of illness were positive for SARS-CoV-2 on RT-PCR which became negative on day 19. This suggests a potential of disease transmission during the course of conjunctivitis. The Ct (cycle threshold) value for detection of SARS-CoV-2 in tear RT-PCR however showed a rising trend with progression in duration of illness, indicating a decrease in tear viral load.^[15]^

The first case of hemorrhagic conjunctivitis with pseudo membrane in COVID-19 patient was reported from France, where ocular manifestations like conjunctival congestion and watery discharge were first noted at day 17 of systemic illness.^[16]^ Direct microscopy/culture of conjunctival swabs and scrapings were done which ruled out any bacterial, chlamydial, and other viral etiology (like Herpes and adenovirus). Ocular condition worsened by day 19, with occurrence of petechiae, follicles, tarsal hemorrhages, chemosis, and formation of a yellowish white pseudo membrane in the lower lids. Examination under fluorescein staining revealed superficial punctate keratitis. RT-PCR of conjunctival swab and scrapings on day 20 was negative for SARS-CoV-2. Daily debridement of membrane was done while patient was started on azithromycin and low-dose dexamethasone, resulting in improvement of signs and symptoms from day 21 to day 26.^[16]^

Furthermore, conjunctivitis can either be the first or only manifestation of COVID-19. This has been highlighted in a case series from Italy where four middle-aged patients (three male and one female) who had recent history of travel to a region with high number of COVID-19 cases presented with acute conjunctivitis.^[11]^ Having a high index of suspicion based on their travel history, nasopharyngeal swabs were taken and sent for RT-PCR which confirmed SARS-CoV-2. They were advised self-quarantine and prescribed topical antibiotics with symptomatic treatment. On follow-up none of them complained of any fever, malaise, or respiratory illness.

In another case report, a 27-year-old male from Argentina who had redness and foreign body sensation in one eye took telephonic consultation from an ophthalmologist. He had lid edema in the same eye with moderate conjunctival hyperemia. There were no systemic complaints at the time of presentation but 12 hr later he developed fever, headache, cough, and severe dyspnea. He was tested positive for SARS Cov-2 on RT-PCR of nasopharyngeal swabs.^[12]^

Thus, there are no hallmark clinical features of COVID-19 conjunctivitis. Watering, itching, dry eyes, and blurring of vision are common presenting symptoms, while major clinical signs include photophobia, conjunctival congestion, chemosis, follicles and preauricular lymphadenopathy.^[20]^ Definitive diagnosis can be established by detection of virus in conjunctival swab samples via RT-PCR.^[21]^ However, reported positivity rate for SARS Cov-2 in conjunctival swab is as low as 5%.^[15]^ Few studies have also reported a positivity rate of 2.5%.^[13,22]^ Hence, diagnosis remains mostly presumptive, in patients with confirmed COVID-19 infection on RT-PCR of nasopharyngeal swabs.

Close examination should be avoided as much as possible in such patients and follow-up should preferably be done through telemedicine.

While examining these patients, all standard precautions and safety guidelines as recommended by American Academy of Ophthalmology^[23]^ should be followed. Slit lamps should be equipped with breath shields or slit-lamp barriers and talking to the patient during examination should be better avoided or kept minimal.^[23]^

Since viral shedding may persist for a prolonged period (longest duration reported in one study is 37 days^[24]^), AAO recommends that a repeat RT-PCR test should be done prior to nonemergency procedures if conducted within six weeks of diagnosing COVID-19 infection.^[23]^

In emergency cases, if patient is COVID-19-positive or status is unknown, use of N95 masks, gowns, face shields, and goggles should be considered. Similarly, patients should wear surgical mask during the procedure.^[23]^

### Sanitizer Aerosol-driven Ocular Surface Disease (SADOSD)

Apart from infection, another common possible cause of conjunctivitis in the COVID era can be the frequent use of sanitizers and hand rubs. This has been discussed in a case report where 26-year-old female complained of recurrent episodes of redness, irritation, and burning sensation in her eyes over past few weeks. On enquiring about any recent change in cosmetics or toiletries she admitted to hourly use of hand sanitizer spray. The authors also reported that 60% of their teleconsultations had been for eye redness, of which only about one-fourth was infective, rest being nonspecific. About 40% were related to healthcare workers.^[1]^

ABHR (alcohol-based hand rub)-induced conjunctivitis may be attributable to associated ocular allergy or desiccation stress induced on ocular surface due to the dehydrating property of alcohol.^[1]^ Also, aerosolized chemicals from sanitizers and disinfectants can adversely affect ocular surface and precorneal tear film.^[25]^

Some preventive measures which can be taken to protect eyes from harmful effects of aerosolized chemicals include judicious use of sanitizers and other disinfectants and ensuring proper ventilation through doors/windows. While spraying, the air-conditioning should be turned off, the bottle nozzle of sanitizer should be below eye level, and eyes to be closed. Hyaluronate-based lubricants should preferably be used for problems related to ocular discomfort and tear film.^[1]^

### Mask and Dry Eye

Wearing a face mask is becoming the current standard. Masks may, however, also cause problems such as intermittent dry eyes,^[26]^ where patient may complain of burning and stinging in eyes after prolonged wearing of face mask. With an increase in the use of oxygen concentrators and approval of CPAP machines in COVID-19 patients with respiratory difficulty, there is also a concern regarding rise in the incidence of dry eyes.^[26]^ Continuous flow of exhaled air due to leakage through the top of the Face/CPAP/oxygen masks is directed straight to our eyes, which can accelerate the evaporation of natural tears. This may cause eye irritation and increased desiccation stress, resulting in inflammation. Hence, promoting normal tear film development and control of inflammation may become vital to prevent dry eyes.

Patients experiencing dry eye symptoms from extended mask wear should take breaks every few hours whenever feasible to take off the mask and reapply lubricant eye drops. Gel-based lubricants can provide long-term symptomatic relief, mitigating the need for frequent instillation of eye drops. Hyaluronic acid-based lubricants can also be used as they not only increase tear retention time but also suppress inflammation.^[27]^

Preference should be given to the masks with a pliable nose-wire, with proper fitting of the shape of the wire to prevent air being directed toward the eyes.^[26]^ Masks may be taped at the top to stop the upward airflow, while taking care that the lower lid movement is not affected.^[26]^

### Digital Eyestrain/Computer Vision Syndrome 

Implementation of lockdown worldwide has compelled people to work from home, even teach and study from home. As outdoor recreational activities are forbidden, people are resorting to digital entertainment. This has resulted in an increased screen exposure time. Eye strain, dry or itchy eyes, headache, blurred vision, physical and mental fatigue are common problems today, even for those who never had them, which is also referred to as computer vision syndrome.^[28]^

To combat these problems, the following steps are advised.

#### Increasing the blink rate 

The normal spontaneous blinking rate of a person varies from 12 to 15/min.^[29]^ However, under relaxed conditions, it has been found up to 22 blinks/min.^[21]^ Blink rate may decrease significantly while reading a book or seeing an electronic device.^[30]^ Less blinking leads to evaporation of tears leading to make the eyes lose moisture and become dry. Thus, the strain on eyes tend to increase.

Hence, the patient should be advised to blink more during the period of screen work. Artificial tears can also be prescribed to reduce the complaint of dryness.

#### Frequent breaks

The 20-20-20 rule should be followed when spending a lot of time looking at a screen. Look 20 feet away for 20 s every 20 min when engaged in screen-related work.^[31]^

#### Proper lighting of surroundings

An equally balanced lighting in the working area not only provides visual comfort but also serves to minimize eyestrain.^[28]^ Excessive sunlight can be filtered with window blinds and tinting.^[28,31]^ Several workplaces have a set of very intense fluorescent lights overhead; in other situations, some of them may be shut off or substituted with lamps full-spectrum or replaced with daylight. Lamps with incandescent bulbs which contain mostly red light (“warmer color”) can also be helpful in preventing eyestrain.^[28]^

#### Minimizing glare

Use of screen filters on computer helps to reduce the reflection, improve contrast, thereby relieving eyestrain.^[28,31]^

#### Wearing the right glasses

Uncorrected hypermetropia, astigmatism, and presbyopia can further contribute to development of eyestrain while using a digital display device.^[31]^ Hence, a proper refractive workup and correction is required which should include cycloplegic refraction.

Contact lenses may further aggravate the symptoms of ocular discomfort caused due to dry eye.^[28]^ It is advised that people wearing contact lenses may switch to glasses when working in front of the computer screen to prevent the irritation and dry eye caused by prolonged use of lenses.

Progressive lenses may be preferred in presbyopes who do jobs related to prolonged computer work. Use of base in and base up prisms can also relieve digital eyestrain by decreasing the effort required in elevation and convergence.^[28]^

#### Adjusting the display settings on the screens you use

This includes display devices like television, computer, and mobile devices. Increasing the type size on computer prevents from leaning over and squinting into the screen. According to a study by Kochurova et al, text size on computer/laptop screen should be at least twice the visual acuity of user for comfortable reading.^[32]^ Screen should be kept clean from dust and fingerprints.^[31]^ Computer display settings like brightness and contrast can be adjusted.

#### Correct ergonomics

It is especially important to maintain sufficient distance from the monitor to prevent the side- effect of prolonged exposure. One should view the smartphone at an angle below the eyebrows and hold the device at a comfortable distance with larger font and brighter screen. Computer users should maintain 20 to 28 inches distance from the monitor with eye level 4 to 5 inches above the center of the screen.^[31]^

### COVID-associated Mucormycosis

COVID patients are at a higher risk of developing opportunistic infections, ${}^{\left[2--5\right]}$ including rhino–orbital mucormycosis ${}^{\left[6--10\right]}$ which is a serious concern for an ophthalmologist as it is a potentially sight-threatening condition.

This higher vulnerability to secondary infections can be attributed to the interplay of various factors like steroid use, systemic comorbidities (like uncontrolled diabetes, malignancy, transplantation), and use of immunomodulatory drugs like tocilizumab and immune dysregulation caused by coronavirus.^[10]^ However, history of steroid use and uncontrolled diabetes were the two most frequently reported risk factors in COVID-associated mucormycosis. ${}^{\left[6--10\right]}$

Rhino–orbito cerebral mucormycosis is an invasive fungal infection involving the nasal cavity, paranasal sinuses, orbit and brain causing high morbidity and mortality. Overall survival rate is reported to be 59.5% with treatment and 21% without treatment.^[33]^ It can also cause serious ophthalmic complications like central retinal artery occlusion resulting in vision loss. Therefore, timely diagnosis and early intervention is of utmost importance. However, absence of reliable diagnostic markers and poor yield on tissue culture make it a diagnostic challenge. Hence, one must have a high index of suspicion especially when patient is not responding to intensive antibiotic therapy and repeated cultures are negative.

Early clinical features are facial pain, periorbital swelling and black eschar over skin overlying the orbit, nasal cavity, and palate. Later orbital involvement may result in ophthalmoplegia, proptosis, ptosis, superior orbital fissure/orbital apex syndrome.^[34]^

Radiological investigations include CT scan and MRI; however, diagnosis is confirmed only on histopathology and culture. In case of repeated cultures, tissue biopsy is gold standard.^[34]^

Management involves control of causative risk factors, systemic antifungal treatment and surgical debridement. Exenteration may be required in case of extensive orbital spread.^[34]^

##  Summary

This COVID-19 pandemic is long to stay. The only method to survive this period is to take all possible precautions. Eye health although often neglected is also getting affected in the current corona crisis. Therefore, following proper hygiene, exercises of the eye, and maintaining the eye health is also important.

##  Financial Support and Sponsorship: 

None.

##  Conflicts of Interest

None.
